# Metabolomic Analysis of Cooperative Adaptation between Co-Cultured *Bacillus cereus* and *Ketogulonicigenium vulgare*

**DOI:** 10.1371/journal.pone.0094889

**Published:** 2014-04-11

**Authors:** Ming-Zhu Ding, Yang Zou, Hao Song, Ying-Jin Yuan

**Affiliations:** 1 Key Laboratory of Systems Bioengineering, Ministry of Education (Tianjin University), Tianjin, PR China; 2 School of Chemical Engineering and Technology, Tianjin University, Tianjin, PR China; 3 Collaborative Innovation Center of Chemical Science and Engineering (Tianjin), Tianjin, PR China; University of Florida, United States of America

## Abstract

The cooperative adaptation of subcultivated *Bacillus cereus* and *Ketogulonicigenium vulgare* significantly increased the productivity of 2-keto-L-gulonic acid, the precursor of vitamin C. The mechanism of cooperative adaptation of the serial subcultivated *B. cereus* and *K. vulgare* was investigated in this study by culturing the two strains orthogonally on agar plates. It was found that the swarming distance of *B. cereus* along the trace of *K. vulgare* on the plate decreased after 150 days' subcultivation. Metabolomic analysis on these co-cultured *B. cereus* and *K. vulgare* strains showed that their cooperative adaptation was accomplished by three key events: (i) the ability of nutrients (e.g., amino acids and purines) searching and intaking, and proteins biosynthesis is increased in the evolved *B. cereus*; (ii) the capability of protein degradation and amino acids transportation is enhanced in evolved *K. vulgare*; (iii) the evolved *B. cereus* was found to provide more nutrients (mostly amino acids and purines) to *K. vulgare*, thus strengthening the oxidation and energy generation of *K. vulgare*. Our results provided novel insights into the systems-level understanding of the cooperative adaptation between strains in synergistic consortium.

## Introduction

Synergistic metabolic cooperation is one of the most important strategies in microbial consortia, including cross-feeding of small molecules (nutrient and energy resources or co-factors), removing by-products, and degradation of complex exogenous compounds [1]–[4]. Microbial cooperation plays an important role for industrial, medical, and biotechnological purposes. The pattern of metabolite exchanges involves tremendous changes during microbial adaptive evolution [4], [5]. An in-depth analysis of the cooperative adaptation in microbial consortia from the perspective of metabolic exchanges could enable a systematic understanding of the complex interactions in symbiotic systems.

There are many co-culture studies that yielded new insights into cooperative interactions [6]–[9]. In the two-step Vitamin C fermentative production processes, *B. cereus* and other *Bacillus spp.* (e.g., *Bacillus subtilis*, and *Bacillus megaterium*) are companion strains, which stimulates the growth of *K. vulgare* and its synthesis of 2-keto-L-gulonic acid (2-KLG). It was found that *B. megaterium* and *K. vulgare* interact by exchanging a number of metabolites, such as amino acids, erythrose, erythritol, guanine, and inositol [8]. Our previous study has reported that subcultivation increased the production of 2-KLG significantly [10]. However, the molecular mechanism of the cooperative adaptation induced by the subcultivation was unclear.

Swarming motility is a multicellular behavior which changed significantly during the subcultivation of co-cultured *B. cereus* and *K. vulgare* in the process of 2-KLG production. To better understand the metabolic interaction and the effects of the cooperative adaptation on swarming motility between the two species, we employed the solid-phase co-culture method that was developed in our previous study [8]. We found that upon cooperative adaption, the swarming distance of *B. cereus* along the trace of *K. vulgare* decreased after 150 days' subcultivation. Metabolomic profiling study was carried out to investigate the metabolic exchanges between the co-cultured *B. cereus* and *K. vulgare* strains (before and after subcultivation) on solid surfaces. Such systematic understanding of the cooperation in the symbiotic ecosystem is of great importance for the optimal production of 2-KLG.

## Materials and Methods

### Strains and cultivation conditions

The *B. cereus* HB601 (specified as B0) and *K. vulgare* HB602 (specified as K0) were separated from soil and donated by Prof. Yuezhong Li, Shandong University, China. The evolved *B. cereus* (specified as B150) and evolved *K. vulgare* (specified as K150) were obtained by 150 days' subcultivation of the B0-K0 co-culture, as detailed in our previous study[10].

The pure mono-cultures including B0, B150, K0, K150 were cultivated at 30°C, 250 rpm, in a 250 ml flask with 50 ml seed medium (2% l-sorbose, 0.3% corn-steep liquor (CSL), 1% peptone, 0.3% yeast extract, 0.3% beef extract, 0.1% urea, 0.1% KH_2_PO_4_, 0.02% MgSO_4_•7H_2_O and 0.1% CaCO_3_) for 48 h, respectively. Then, B0, B150, K0, K150 were co-cultured in solid sorbose-CSL medium containing 1.7% agar and cultivated at 30°C for 96 h. The experiment was designed as the pattern in Fig. S1. Cells of K0 and K150 were first cultured on the yellow place of the plate horizontally, respectively. Then, B0 and B150 were cultured vertically on the blue place of the plate, respectively (Fig. S1).

### Sampling, quenching, extraction and derivatization of metabolites

*B. cereus* and *K. vulgare* were mono-cultured and co-cultured in a plate, respectively. The co-cultured cells were sampled at the overlapping point (specified as B0K0, B150K0, B0K150, and B150K150) as shown in the red square in Fig. S1. The mono-cultured and co-cultured cells were spaded from the 1 cm×0.6 cm agar surface with 0.5 ml cold ultrapure water twice at 48 h and 96 h, respectively. The mixture was centrifuged at 5000 g for 3 min. The supernatant was prepared as the extracellular metabolites sample. The precipited cells were quenched and extracted as intracellular metabolites according to our previous method [11]. An extra group of quenched cells was washed and dried to calculate the dry weight of the sampled cells. To correct for minor variations occurring during sample preparation and analysis, 10 µl succinic *d_4_* acid (0.1 mg/ml) was used as an internal standard. The extracts of intracellular (100 µl) and extracellular (20 µl) metabolites were both lyophilized. Four independent experiments were performed for each sample.

Two-stage chemical derivatization was performed as described previously [11]. Firstly, methoximation of the carbonyl groups was carried out by dissolving sample in 50 µL methoxamine hydrochloride (20 mg/mL in pyridine) and incubating it at 40°C for 80 min. Then, 80 µL *N*-methyl-*N*-(trimethylsilyl) trifluoroacetamide (MSTFA) was added and it was incubated at 40°C for 80 min for trimethylsilylation.

### Metabolomic analysis by GC-TOF/MS

Intracellular and extracellular metabolites were analyzed by GC-TOF/MS (Waters Corp., USA) as described previously [11]. One microliter of derivatized sample was injected by Agilent 7683 autosampler into GC (Agilent 6890) which was equipped with DB-5MS column (30 m×0.25 mm×0.25 µm, J&W Scientific, Folsom, CA). The oven temperature was programmed as: 70°C for 2 min, then increased to 290°C (5°C/min), holding for 3 min. The ion source temperature and ionization current were 250°C and 40 µA, respectively. The mass scan range was 50–800 m/z.

Peak detection, deconvolution, and peak quantification were performed using Masslynx software (Version 4.1, Waters Corp., USA) [12]. Metabolites were identified by comparing their mass fragmentation patterns with NIST mass spectral library. The area of each acquired peak was normalized against that of internal standard and dry cell weight for calculating the relative abundance of each metabolite.

The relative abundance of metabolites, after normalized by centering and pareto scaling, were analyzed by partial least-square discriminant analysis (PLS-DA) using SIMCA-P +11.5 software (Urimetrics AB, Umea, Sweden). Four biological replicates were used to perform multivariate analysis for each sample. To present the variations of metabolites in heatmap, the relative levels of these metabolites were standardized by mean 0 and variance 1, and then clustered using Expander 4.1 (EXpression Analyzer and DisplayER).

## Results

### Decreased swarming motility of evolved *B. cereus*

**Figure 1:**
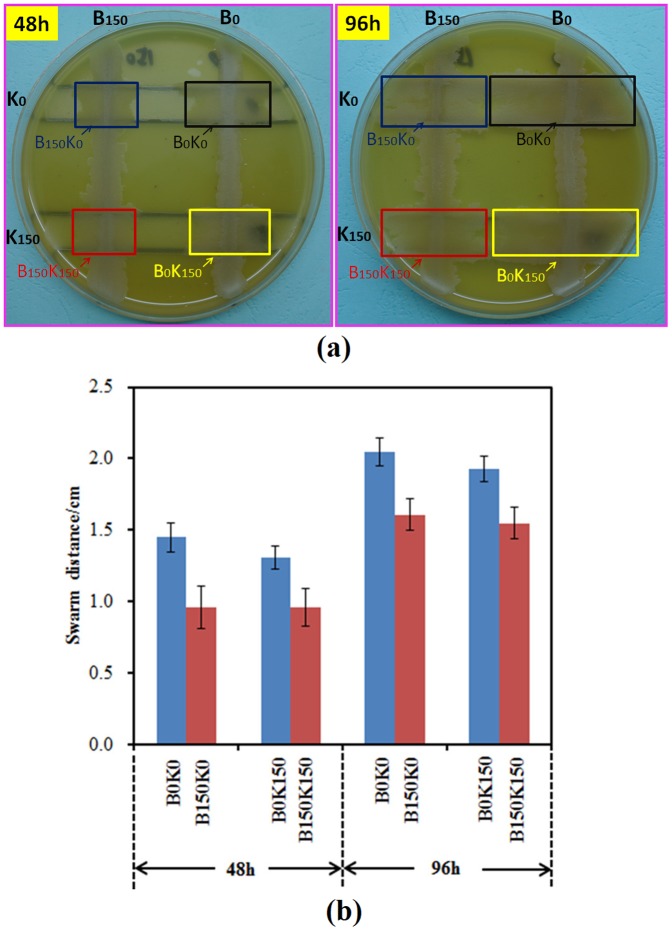
Swarming motility of *B. cereus* co-cultured with *K. vulgare* on agar plate. (a) Pictures of swarming motility in 0^th^ and 150^th^ day's co-cultured *B. cereus* and *K. vulgare* at 48 h and 96 h; (b) Swarming distance of *B. cereus* co-cultured with *K. vulgare* on soft agar plate before and after 150 days' subcultivation.

Swarming motility of *B. cereus* could be induced when it was co-cultured with *K. vulgare* on an agar plate. In the co-culture with either K0 or the evolved K150, the swarming distance of B150 was all significantly reduced in comparison to B0, as demonstrated in Fig. 1. It indicated that the swarming motility of the evolved *B. cereus* was weakened, and the cooperation between *B. cereus* and *K. vulgare* changed upon 150 days' subcultivation. Our previous study [10] reported that the serial subcultivation of *B. cereus* and *K. vulgare* increased the yield of 2-KLG from 77% (original co-culture, B0K0) to 93% (evolved co-culture, B150K150) in liquid medium. It could be speculated that the metabolite exchange between the co-cultured *B. cereus* and *K. vulgare* varied significantly due to the cooperative adaption. The present study further investigated the evolved cooperation of *B. cereus* and *K. vulgare* in a systematic way *via* metabolomic profiling.

### Metabolomic profiling

Both the intracellular and extracellular metabolites of the co-cultured *B. cereus* and *K. vulgare* strains (before and after subcultivation) were analyzed by GC-TOF/MS to identify the metabolic exchanges during the adaptive evolution. Metabolomic profiling reflected many differences in the intracellular metabolism between B0K0, B150K0, B0K150 and B150K150, as well as B0, B150, K0 and K150. The increased (ratio>2.0) and decreased (ratio<0.5) metabolite numbers were normalized to the total metabolite number in mono-cultured and co-cultured samples, respectively. It was shown in Fig. S2 that more than 90% intracellular metabolites increased in the co-cultures (i.e., B150K0/B0K0 and B150K150/B0K150) at 48 h. It indicated that most metabolites in B150 increased significantly after subcultivation when it was co-cultured with K0 or K150. However, for both mono-culture of wild-type (B0 and K0) and subcultivated *B. cereus* or *K. vulgare* (B150 and K150), less than 20% and 10% metabolites increased, respectively (Fig. S2). Thus, B150 exhibited better metabolic activity than B0 when co-cultured with *K. vulgare* that B150 did not need to swarm further to get more nutrition (Fig. 1). The changes of the extracellular metabolites were not as significant as the intracellular metabolites upon subcultivation. The most significant change was that more than 70% metabolites increased in the medium of K150 compared to K0 (Fig. S3).

**Figure 2:**
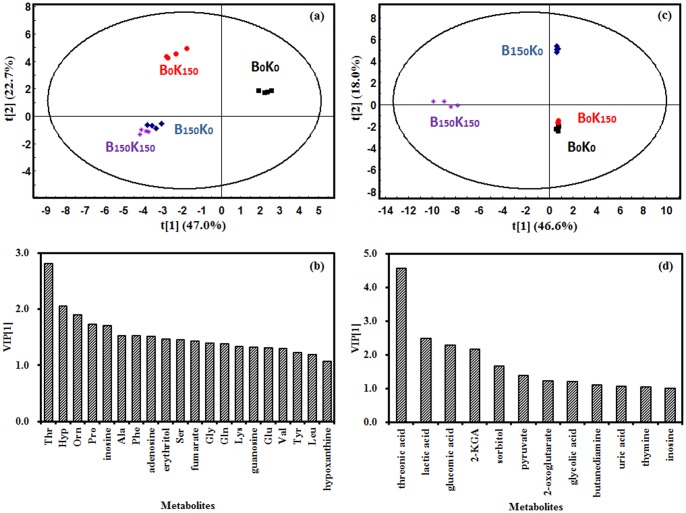
PLS-DA plots of intracellular samples in co-cultured *B. cereus* and *K. vulgare*. (a) score plot of t[1]/t[2] of samples at 48 h; (b) VIP plot generated from the metabolic data of 48 h; (c) score plot of t[1]/t[2] of samples at 96 h. (d) VIP plot generated from the metabolic data of 96 h. The explained variance was in brackets. The ellipse defines the Hotelling's T2 confidence region (95%). The VIP[1] values represent the contributing weights of each metabolite to 1^st^ principal component of the PLS-DA model.

PLS analysis was carried out on intracellular metabolites of co-cultured B0K0, B150K0, B0K150, and B150K150 samples at 48 h (Fig. 2a) and 961h (Fig. 2c). We found that the sample points between B0K0 and B150K0, B0K150 and B150K150 were far away from each other at both 48 h and 96 h, which indicated distinctive metabolism characteristics between B0K0 and B150K0, as well as B0K150 and B150K150.

A total of 20 and 12 variables contributed to the discriminating PLS model (VIP value >1) of samples at 48 h and 96 h, respectively (Fig. 2b and 2d). It was found that amino acids (e.g., Thr, Orn, Pro, and Ala), nuclosides (inosine, adenosine, and guanosine), purines (hypoxanthine), TCA cycle intermediates (e.g., fumarate), and polyols (e.g., erythritol) were of crucial importance in the sample discrimination at 48 h. PLS analysis at 96 h showed that oxidation related organic acids (e.g., threonic acid, lactic acid, 2-KLG, pyruvate, and glycolic acid) displayed significant differences among each sample.

### Metabolic changes of co-cultured *B. cereus* and *K. vulgare* upon subcultivation

#### Amino acids metabolism

**Figure 3:**
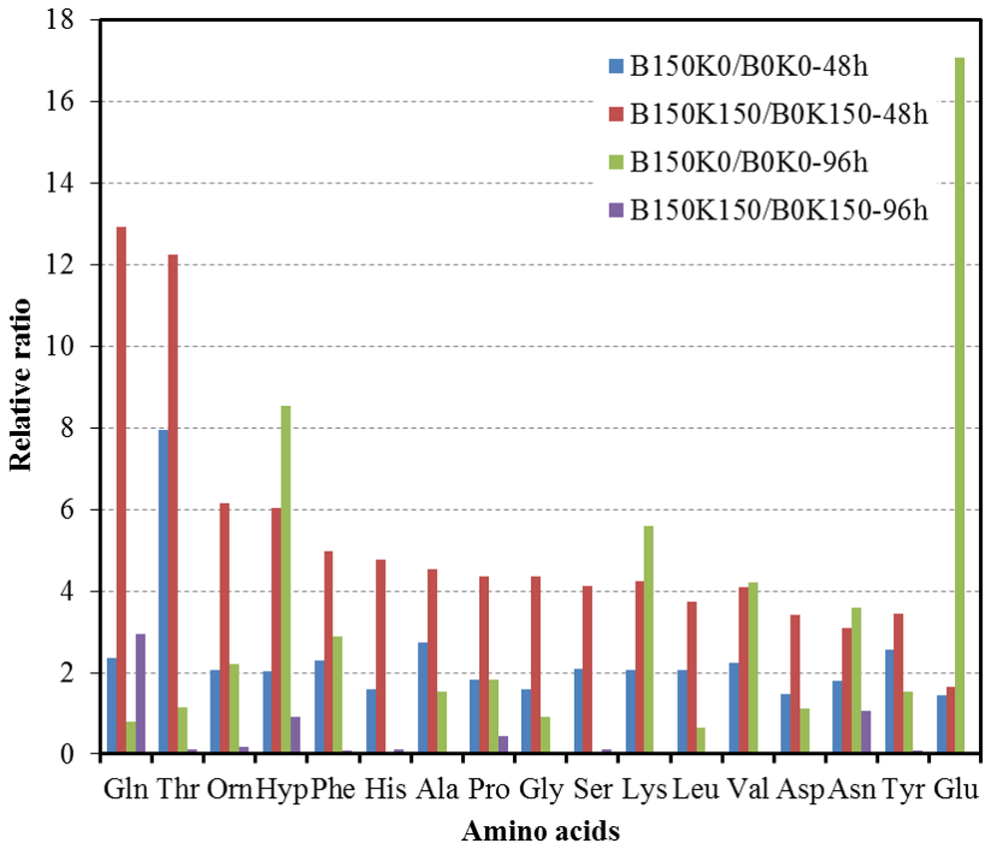
Changes of identified intracellular amino acids before and after 150 days' subcultivation in co-cultured *B. cereus* and *K. vulgare*. The y-axis was relative ratio. B150K0/B0K0 means the relative ratio of co-cultured B150K0 to co-cultured B0K0. B150K150/B0K150 means the relative ratio of co-cultured B150K150 to co-cultured B0K150. Each value represented mean value of four independent replicates. Gln: glutamate; Thr: threonine; Orn: orinine; Hyp: hydroxyproline; Phe: phenyalanine; His: histidine; Ala: alanine; Pro: proline; Gly: glycine; Ser: serine; Lys: lysine; Leu: leucine; Val: valine; Asp: aspartic acid; Asn: asparagine; Tyr: tyrosine; Glu: glutamic acid.

The metabolic differences of co-cultured *B. cereus* and *K. vulgare* before and after 150 days' subcultivation were investigated. One of the most specific variations was the increase in amino acids metabolism when B150 was co-cultured with either K0 (B150K0) or K150 (B150K150) comparing to the co-cultures containing B0 (B0K0 or B0K150) (Fig. 3). The levels of amino acids in the co-culture showed that the ratio of B150K150/B0K150 was higher than B150K0/B0K0 at 48 h, and both ratios were larger than 1 (Fig. 3). It indicated that the abundance of most amino acids in *B. cereus* increased significantly upon subcultivation with *K. vulgare.* PLS analysis also validated this result that amino acids played important roles in distinguishing B150K0 with B0K0, as well as B150K150 with B0K150 (Fig. 2c). However, the swarming motility did not show much difference between B150K150 and B150K0, as well as B0K0 and B0K150 (Fig. 1a), which demonstrated that swarming motility was only one result caused by the adaptive cooperation between *B. cereus* and *K. vulgare*.

At 96 h, the level ratios of most amino acids in B150K0 to B0K0 were larger than 1, while those in B150K150 to B0K150 were smaller than 1 (Fig. 3), which indicated that most amino acids increased in B150K0 relative to B0K0, whereas decreased in B150K150 relative to B0K150. This suggested that amino acids were dramatically consumed at 96 h in B150K150, which might be used to synthesize proteins for better growth and cooperation of the co-culture. Most intracellular amino acids (e.g., Gln, Thr, Phe, and Asn) increased in B0 from 48 h to 96 h, while all decreased in B150 (Fig. 3). All extracellular amino acids decreased in both B150 and B0 at 96 h comparing with that of 48 h. The changes in B150 were more significant than that in B0 (E-B150 > E-B0, Fig. S3a), indicating B150 used more amino acids to synthesize proteins for its better growth. In addition, the consumption of extracellular amino acids in the co-culture increased after subcultivation of *B. cereus* (E-B150K0 > E-B0K0, E-B150K150 > E-B0K150, Fig. S3b), which further indicated that more amino acids were needed for evolved cells to synthesize proteins. This was in accordance with the changes of intracellular amino acids.

#### Purine and nucleoside biosynthetic pathway

**Figure 4:**
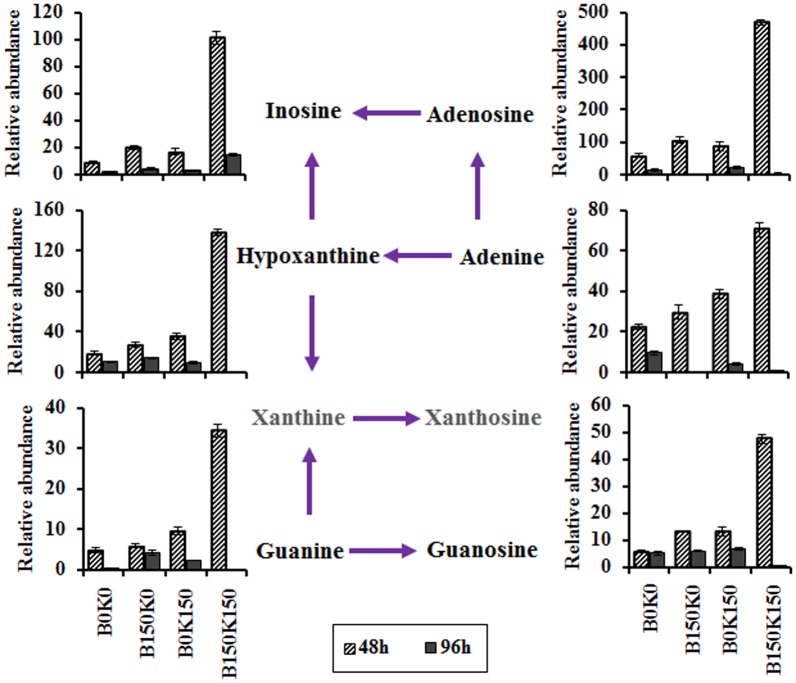
Changes of intracellular metabolites in purines and nucleosides biosynthetic pathway in the consortium. The y-axis was the relative abundance, being calculated by normalization of peak area of each metabolite to internal standard and dry weight of cells. Each value represented mean value of four independent replicates, and the error bars show the standard deviations.

The levels of intracellular purines (guanine, hypoxanthine, and adenine) and nucleosides (inosine, adenosine, and guanosine) in B150K150 and B150K0 were higher than that in B0K150 and B0K0 at 48 h, respectively (Fig. 4). In particular, these metabolites in B150K150 were much higher than that in B0K150, which demonstrated that B150 accumulated high levels of purines and nucleosides to synthesize nucleic acids when co-cultured with K150. At 96 h, all the detected intermediates in purine and nucleoside biosynthetic pathway decreased significantly, especially in B150K150 (Fig. 4), which might be caused by the much faster synthetic rate of nucleic acid in the consortium. Thus, nucleic acids biosynthesis was enhanced by subcultivation of the consortium.

#### Glycolysis and TCA cycle

**Figure 5:**
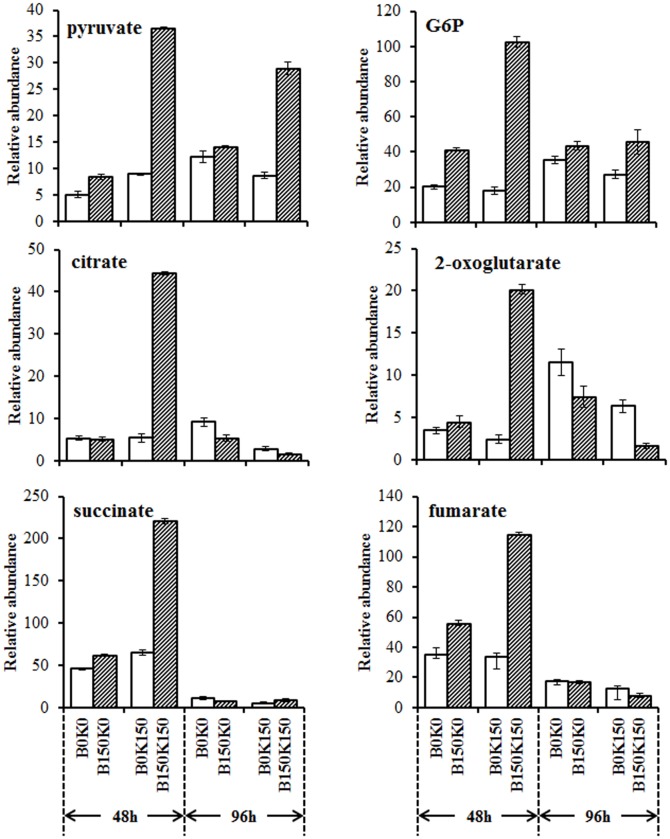
Changes of glycolysis and TCA cycle intermediates. The y-axis was the relative abundance being calculated by normalization of peak area of each metabolite to internal standard and dry weight of cells. Each value represented mean value of four independent replicates, and the error bars show the standard deviations.

The levels of most detected metabolites in glycolysis and TCA (including pyruvate, G6P (glucose-6-phosphate), citrate, 2-oxoglutarate, fumarate, and succinate) increased significantly in the co-cultured *B. cereus* and *K. vulgare* after subcultivation at 48 h (Fig. 5), in particular in B150K150. This result suggested that the evolved B150 demonstrated more active metabolic activity in glycolysis and TCA cycle than that in B0 when co-cultured with *K. vulgare* (K0 or K150). Furthermore, the K150 co-cultured consortia (B0K150 and B150K150) demonstrated much higher difference in the level of these metabolites in glycolysis and TCA than that in K0 co-cultured consortia (B0K0 and B150K0), which indicated that K150 exerted more severe effect on the cooperation.

The changes of pyruvate and G6P at 96 h were similar to those at 48 h. However, the levels of other four TCA intermediates in B150K150 and B150K0 were lower than or similar to these in B0K150 and B0K0, respectively, which might be caused by the different sporulation of the parental and evolved *B. cereus.*

#### Stress and oxidation related metabolites

**Figure 6:**
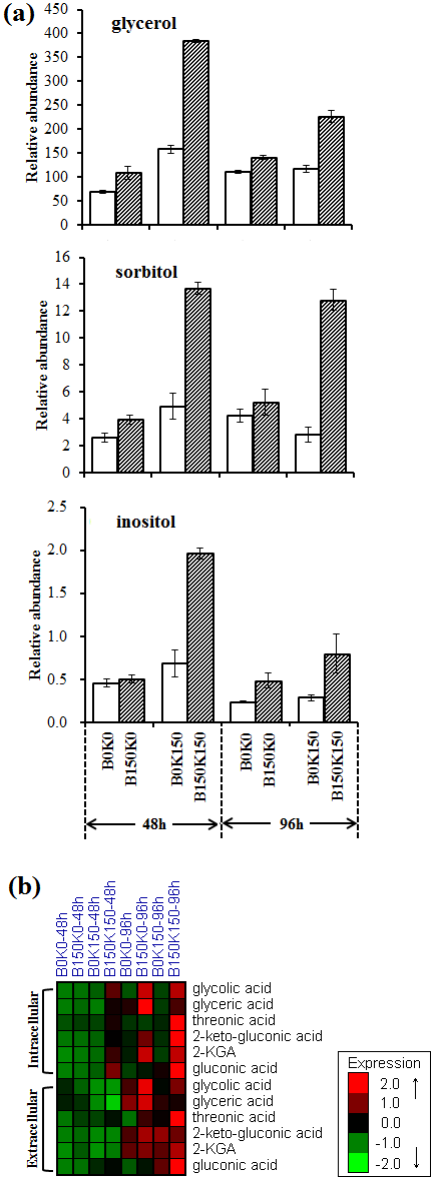
Changes of (a) stress related polyols and (b) oxidation related metabolites. Each value represented mean value of four independent replicates, and the error bars show the standard deviations.

Intracellular metabolites including glycerol, inositol and sorbitol were reported to be closely related to stress defense of bacteria. Here, we found that these intracellular metabolites were all in higher level in the co-culture of B150K150 and B150K0 than in B0K150 and B0K0, respectively (Fig. 6a). It indicated that the evolved B150 displayed more significant defense ability against stress when co-cultured with *K. vulgare*. However, the increase in these intracellular metabolites in *B. cereus* (i.e., B150 vs. B0) co-cultured with K150 was more dramatic than that co-cultured with K0, which suggested that when the evolved K150 was co-cultured with the evolved B150, the ability of its stress resistance was dramatically strengthened.

Organic acids (including 2-KLG, 2-keto-gluconic acid, gluconic acid, glycolic acid, threonic acid, and glyceric acid) were oxidative products of *K. vulgare*. These oxidation related metabolites (intracellular and extracellular) displayed higher level in B150K150 and B150K0 than in B0K150 and B0K0, respectively (Fig. 6b). It indicated that the oxidative ability of *K. vulgare* increased when it was co-cultured with B150 than that with B0. In addition, the oxidative ability of K150 was higher than K0 when co-cultured with B0 or B150.

## Discussion

**Figure 7:**
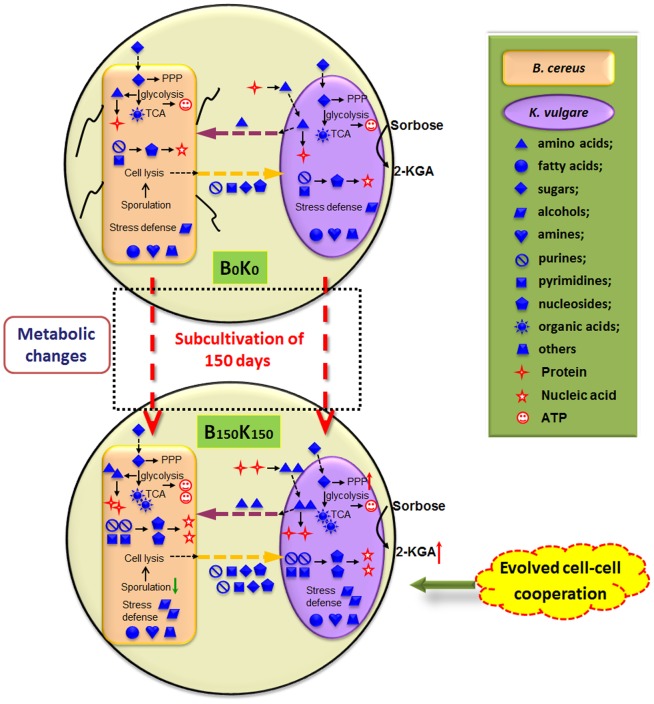
Schematic depicting the possible mechanism for evolved metabolic cooperation between *B. cereus* and *K. vulgare*.

In our previous study, the investigation on the metabolic cooperation in the co-culture system of *B. megaterium* and *K. vulgare* revealed that the interaction was a synergy of mutualism and antagonism [8]. However, the cooperation between *B. cereus* and *K. vulgare* undergoes a significant change after 150 days' subcultivation, and their metabolites exchange is enhanced. It was speculated that the relationship between the adapted *B. cereus* and *K. vulgare* changed to mutualism [13], i.e., they can provide the required nutrients to each other in an enhanced manner. The metabolic exchanges between *B. cereus* and *K. vulgare* before and after subcultivation were depicted in Fig. 7. The adaptive cooperation is accomplished by the following crucial events. On the one hand, the adapted B150 acquired enhanced capability than the parental B0 in nutrients' (e.g., amino acids and purines) intake and transportation, proteins biosynthesis from amino acids, membrane permeability, and stress tolerance to harmful metabolites. On the other hand, the adapted K150 acquired more capability compared to the parental K0 in protein degradation to amino acids and the intake of amino acids, which increased the growth and production of 2-KLG by K150. Apart from metabolite exchanges, evolved *B. cereus* assisted *K. vulgare* in combating ROS more efficiently [14], [15].

### Increased metabolism of the consortium caused by cooperative adaptation

B150 possessed enhanced capability of nutrients and energy intake, which was reflected by the higher levels of intracellular amino acids, purines, glycolysis and TCA intermediates in the co-culture with B150 (B150K0 and B150K150) than in that with B0 (B0K0 and B0K150), respectively. It was found in our previous report that the lag phase shortened, and the growth rate and final biomass increased in B150 [10]. We found here that the adapted B150 enhanced the oxidation ability of *K. vulgare*. These findings indicated that B150 had adapted to the culture environment and could utilize the nutrients more rapidly, which might be one of the reasons that caused the weakened swarming motility when cooperated with *K. vulgare* on agar plate. Furthermore, the membrane permeability was decreased in the adapted *B. cereus* (our unpublished data) that the nutrients provided for the growth of *B. cereus* increased.

Purine nucleotides biosynthesis were reported to be insufficient in *K. vulgare* [16]. Purines (such as guanine and adenine) from the media or the lysis of *B. megaterium* played a vital role in enhancing the growth and metabolism of *K. vulgare* [17], and the addition of adenine, guanine, xanthine, and hypoxanthine could improve the growth of *K. vulgare* [17], [18]. In this study, the increased intracellular levels of purines (guanine, hypoxanthine, and adenine) and nucleosides (inosine, adenosine, guanosine) in the consortium of B150 co-cultured with *K. vulgare* suggested that B150 provided more purines and nucleosides to K150 than that before subcultivation. It further indicated that B150 did not need to swarm as far as B0 to get enough nutrients, which validated the result that the swarming motility of B150 was weakened after 150 days' subcultivation.

Protein synthesis was of great importance for growth, metabolism and reproduction of cells. Thus, biosynthesis of more proteins in *B. cereus* that can feed to *K. vulgare* was necessary. In this study, we found the consumption of amino acids in the medium of B150 was higher than that of B0, which further verified that the adapted *B. cereus* could enhance the growth of *K. vulgare*.

The requirement to conserve energy was an important feature of stress responses. In this study, one of the most pronounced changes in the evolved co-culture was the increase in the metabolites in glycolysis and TCA cycle. The increase in most measured metabolites in TCA cycle and glycolysis after subcultivation were in agreement with the energy conservation strategy of cells. Global gene expression profile for swarming *B. cereus* already showed that swarming caused down-regulation of genes for energy production [19]. Thus, the weakened swarming motility of B150 in the consortium was an important strategy to conserve its energy for the adaptive evolution.

### Weakened swarming motility of evolved *B. cereus* caused by metabolic exchanges

Swarming motility is a cooperative migration powered by rotating flagella, which is crucial for bacteria to seek nutrients [20], [21]. It would increase metabolic cost for the enhanced flagellum synthesis [22]. Thus, the nutrient availability is an important factor for bacterial swarming. The 150 days' subculture caused significantly increased growth and 2-KLG production of *K. vulgare* [10], whereas the swarming motility of *B. cereus* was weakened (Fig. 1). Therefore, the weakened swarming motility of B150 might be due to the increased capability of nutrients intake of the evolved *B. cereus.* Nutritional signal is an important factor in initiating the sporulation of cells [23], [24]. In response to starvation for sources of carbon, nitrogen, or phosphorus, sporulation can be initiated in *B. megaterium* [25]. Sporulation of B150 was less than B0 [26] in the co-culture, which might be due to enhanced capability of nutrition intake in B150. Thus, the swarming motility of B150 in the co-culture reduced comparing with B0. After the 150 days' subcultivation of the co-culture, the metabolic exchanges between *B. cereus* and *K. vulgare* acquired significant changes, which led to better metabolism activity of B150 compared to B0 (Fig. 7). The nutrients in the co-culture containing B150 was sufficient to repress its sporulation [23], which was further validated by the enhanced capability of amino acid and purine intake in B150. The supply of proteins, purines, pyrimidines and small molecules to promote the growth of *K. vulgare* would stop when *B. megaterium* was in sporulation phase [27], [28]. The enhanced nutrient supply (e.g., amino acids, purines) after adaptation thus supported the decreased sporulation in B150. It was reported that sporulation apparently depended on the synthesis of highly phosphorylated nucleotides in *B. subtilis* [29]. Higher levels of purines and nucleosides in the co-culture containing B150 suggested that lower levels of nucleosides were used for sporulation of B150.

On the other hand, swarming is a high energy-consuming process that requires synthesis of many flagella [22], and depends on the constant availability of energy [29]. The weakened swarming motility of B150 in the consortium suggested that the energy consumption decreased in B150. Consequently, the decreased sporulation of B150 would help to save energy to defend the stress in the co-culture. Polyols was reported to play important roles in stress resistance, and it accumulated in yeast in response to inhibitors [11] and ethanol stress [30]. The increased stress defense related polyols in the evolved co-culture suggested that the consortium possessed better stress tolerance. Thus, B150 did not need to form spores as much as B0 to defend the stresses during its growth. The reduced sporulation of B150 indicated that higher metabolism activity was obtained for the subcultivation and it would result in more efficient metabolic exchange with *K. vulgare*.

In a word, it was found that the evolved *B. cereus* could provide more nutrients to *K. vulgare* for its enhanced growth. Meanwhile, the nutrients' intake and transportation capability, the amino acid transport and metabolism, the oxidation ability and stress response in *K. vulgare* were strengthened. Thus, the production of 2-KLG in *K. vulgare* was significantly increased after 150 days' subcultivation. Upon evolution *via* subcultivation, the interaction between *B. cereus* and *K. vulgare* changed from synergistic mutualism and antagonism in the wild-type co-culture to mutualism in the evolved co-culture. These results would contribute to the systematic understanding of the cooperation between evolved *B. cereus* and *K. vulgare* for the higher productivity of 2-KLG.

## Supporting Information

Figure S1 **Experiment design.** Cells were sampled at the overlapping point (specified as B0K0, B150K0, B0K150, and B150K150) as shown in the red square.
(TIF)

Figure S2 Percent of significantly increased (change fold>2) and decreased (change fold<0.5) intracellular and extracellular metabolites after evolution.
(TIF)

Figure S3 (a) log scale of the relative abundance of amino acids in *B. cereus* at 96 h compared to that at 48 h. E-B150 and E-B0 means the relative abundance of extracellular amino acids in B150 and B0, respectively; B150 and B0 means the relative abundance of intracellular amino acids in B150 and B0, respectively (b) log scale of the relative abundance of extracellular amino acids levels in each consortium at 96 h compared to that at 48 h.
(TIF)
