# Supplementary figures and images for: Metabolomic Analysis of Cooperative Adaptation between Co-Cultured Bacillus cereus and Ketogulonicigenium vulgare
Source: PLoS One. 2014 Apr 11;9(4):e94889. doi: 10.1371/journal.pone.0094889 (PMC3984275; doi:10.1371/journal.pone.0094889)

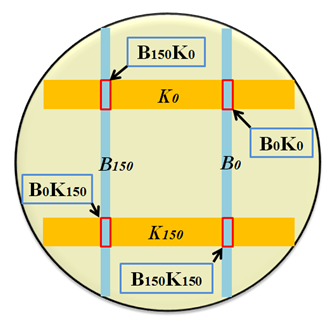

Supplement: Figure S1 — Experiment design. Cells were sampled at the overlapping point (specified as B0K0, B150K0, B0K150, and B150K150) as shown in the red square. (TIF) [file pone.0094889.s001.tif]

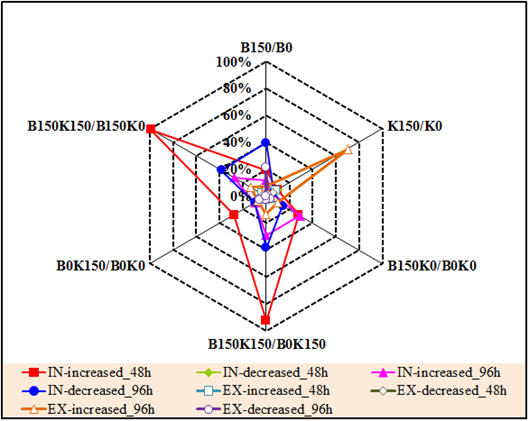

Supplement: Figure S2 — Percent of significantly increased (change fold>2) and decreased (change fold<0.5) intracellular and extracellular metabolites after evolution. (TIF) [file pone.0094889.s002.tif]

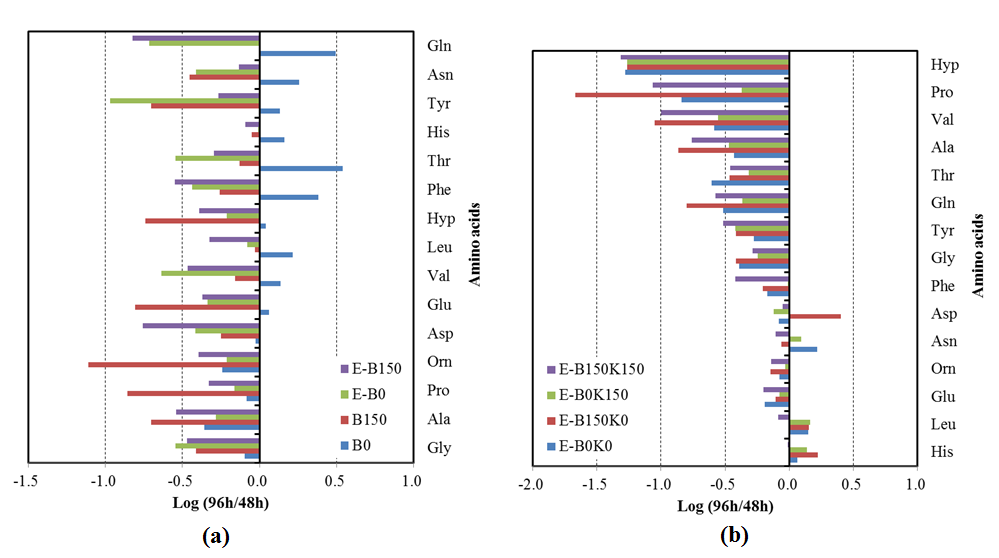

Supplement: Figure S3 — (a) log scale of the relative abundance of amino acids in B. cereus at 96 h compared to that at 48 h. E-B150 and E-B0 means the relative abundance of extracellular amino acids in B150 and B0, respectively; B150 and B0 means the relative abundance of intracellular amino acids in B150 and B0, respectively (b) log scale of the relative abundance of extracellular amino acids levels in each consortium at 96 h compared to that at 48 h. (TIF) [file pone.0094889.s003.tif]
